# Brain lesion extent, growth, and body composition in children with cerebral palsy

**DOI:** 10.1111/dmcn.16427

**Published:** 2025-07-31

**Authors:** Stina Oftedal, Simona Fiori, Kristie L. Bell, Katherine A. Benfer, Leanne Sakzewski, Robert S. Ware, Peter S. W. Davies, Roslyn N. Boyd

**Affiliations:** ^1^ Queensland Cerebral Palsy and Rehabilitation Research Centre, Faculty of Health, Medicine and Behavioral Science The University of Queensland Brisbane Queensland Australia; ^2^ Neuroscience and Medical Genetics Department, Meyer Children's Hospital IRCCS University of Florence Florence Italy; ^3^ Children's Health Queensland Hospital and Health Service South Brisbane Queensland Australia; ^4^ Griffith Biostatistics Unit Griffith University Brisbane Queensland Australia; ^5^ Child Health Research Centre The University of Queensland Brisbane Queensland Australia

## Abstract

**Aim:**

To investigate the relationship between growth, body composition, and the extent of brain lesion measured using structural magnetic resonance imaging (MRI) in children with cerebral palsy (CP).

**Method:**

This prospective population‐based cohort study recorded 359 assessments from 124 children with CP aged 18 months to 13 years (38% female, Gross Motor Function Classification System [GMFCS] levels I = 50, II = 24, III = 17, IV = 12, and V = 21). A neurologist assessed the extent of the brain lesion using a validated semi‐quantitative scale (global, basal ganglia/brainstem, hemispheric and corpus callosum scores). Height (HTZ), weight (WTZ), and head circumference (HDZ) z‐scores were calculated. The Fat Mass Index (FMI) and Fat‐Free Mass Index (FFMI) were determined using a deuterium dilution technique, bioelectrical impedance or dual‐energy X‐ray absorptiometry, and height. Data were analysed using mixed‐effects linear regression.

**Results:**

Greater global (*β* = −0.04, 95% confidence interval [CI] = −0.07 to −0.02), basal ganglia/brainstem (*β* = −0.06, 95% CI = −0.11 to −0.02), corpus callosum (*β* = −0.27, 95% CI = −0.27 to −0.12), and hemispheric (*β* = −0.08, 95% CI = −0.12 to −0.04) scores were associated with lower HTZ. Greater global (*β* = −0.03,95% CI = −0.06 to −0.01) and corpus callosum (*β* = −0.23, 95% CI = −0.40 to −0.06) scores were associated with lower WTZ. A greater hemispheric score (*β* = −0.06, 95% CI = −0.119 to −0.001) was associated with lower HDZ. Semi‐quantitative MRI scores were not associated with FMI or FFMI.

**Interpretation:**

Greater extent of the brain lesion was significantly associated with lower HDZ, HTZ, and WTZ but not body composition in children with CP aged 18 months to 13 years.

## Graphical abstract

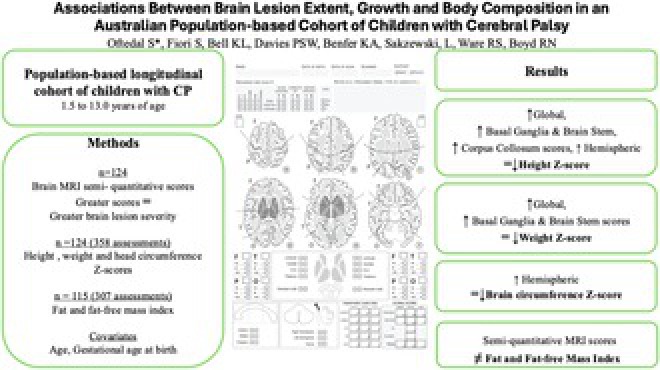

**Abbreviations**
DAG: directed acyclic graph
EDACS: Eating and Drinking Abilities Classification System
FFMI: Fat‐Free Mass Index
FMI: Fat Mass Index
HDZ: head circumference z‐score
HTZ: height z‐score
MRICS: Magnetic Resonance Imaging Classification System
sqMRI: semi‐quantitative magnetic resonance imaging
WTZ: weight z‐score

**What this paper adds**

- The extent of the brain lesion was associated with growth but not body composition.
- Greater global score and all subscores were associated with shorter height.
- Greater global and basal ganglia/brainstem scores were associated with lower weight.
- Greater hemispheric score was associated with a smaller head circumference.
- Brain lesion scores were not associated with the Fat Mass Index or Fat‐Free Mass Index.

Altered growth and body composition, and associations with age, gestational age at birth, feeding difficulties, feeding mode, and level of gross motor impairment in children with cerebral palsy (CP) have been well documented.^1^, ^2^, ^3^, ^4^, ^5^ A low weight z‐score (WTZ) has been associated with increased morbidity and mortality in children with CP across all levels of motor ability.^5^ Importantly, these relationships are associative only; cause and effect have not been determined. The aetiology of poor growth in children with CP has been the subject of many investigations. Malnutrition, endocrine dysfunction, reduced mechanical force, and neurological impacts have been identified as contributing to poor growth in every dimension, including height, body weight, muscle and bone mass, and altered body fat.^6^, ^7^, ^8^, ^9^, ^10^, ^11^

CP is an early‐onset, lifelong neurodevelopmental condition characterized by limitations in activity because of impaired development of movement and posture.^12^ It occurs from maldevelopment attributed to dysplasia of, or injury to, the fetal or infant brain that is not degenerative, although manifestations may change with age.^12^ Neuroimaging studies have furthered our understanding of the aetiology and pathogenesis of CP;^13^ however, the associations between the nature of the brain injury and physical growth and body composition of children with CP is yet to be investigated. Until recently, classification of brain injury has relied on qualitative determination of lesion types using the Magnetic Resonance Imaging Classification System (MRICS).^14^ Development of a reliable and validated visual, semi‐quantitative scale allows quantification of the brain injury for individuals with CP using structural magnetic resonance imaging (MRI) according to extent and location.^15^, ^16^, ^17^ This may allow further understanding of how the extent of the brain lesion and its location influence growth and body composition using quantitative neuroanatomical characterization, as there is large variability within the broad MRICS groups. The primary aim (aim 1) of this study was to investigate the longitudinal relationship between the extent of the brain lesion assessed on structural MRI using semi‐quantitative MRI (sqMRI) scores, growth, and body composition in a population‐based sample of preschool‐age children with CP. The secondary aim (aim 2) was to describe differences in growth and body composition between children with CP and typically developing children (reference population) according to Gross Motor Function Classification System (GMFCS) level, after adjusting for the extent of the brain lesion.

## METHOD

### Participants

Children were recruited into two longitudinal population‐based cohort studies, that is, a Queensland CP Child study of (1) brain structure and motor development and (2) growth nutrition and physical activity. Children were recruited to the first study if they were born between 1st January 2006 and 31st December 2009 in the state of Queensland, Australia and had a confirmed diagnosis of CP. From this group, children born after 1st September 2006 were recruited to the second study. Recruitment and assessment occurred between April 2009 and March 2015. Children were then followed up for a once‐off appointment between 8 years and 12 years in the PREDICT‐CP study^18^ between October 2015 and October 2020. Participants were excluded if they had a progressive or neurodegenerative lesion or chromosomal abnormality known to affect growth and not historically considered CP. Participants were recruited through the Children's Health Queensland Hospital and Health Service and other regional hospitals and health service districts throughout Queensland. Studies were registered with the Australian New Zealand Clinical Trials Registry (nos. 1261200169820, 12611000616976, and 12616001488493).

Written informed consent was obtained from the parents or legal guardians. Ethical approval was received from the University of Queensland and the Children's Health Services District Ethics Committees and eight other sites.^18^, ^19^, ^20^ Children and parents or guardians attended their closest centre or outreach hub location for the assessments. The same study team visited the different geographical locations to collect data. Assessments for the growth, nutrition, and physical activity study primarily occurred on three occasions: at 18 months or 2 years of age; at 3 years of age; and finally at 5 years of age. Children diagnosed after 25 months of age entered the study late. Growth and body composition for the CP Child study sample has been reported previously without adjusting for the extent of the brain lesion.^21^, ^22^

### Measurements

Structural MRI was obtained through the Royal Children's Hospital, Brisbane Medical Imaging Department on a GE Signa Echo Speed 1.5 T MR scanner. The minimum imaging protocol for patients with suspected CP consisted of axial fast spin echo and coronal fast spin echo sequences and three‐dimensional inversion‐prepared fast spoiled gradient recalled acquisition in the steady state sequence. Three‐dimensional acquisitions were reformatted in the axial, coronal, and sagittal planes. Age‐specific protocols were used to maximize the ability to detect cortical and white matter abnormalities at distinct stages of myelination.

The extent of the brain lesion was classified using an sqMRI score by the developer (SF), based on visual assessment of the brain lesion site and its extent.^15^, ^16^, ^17^ Three subscores (hemispheric, basal ganglia/brainstem, and corpus callosum) and a global score were calculated (possible range: 0–48). The hemispheric score describes the extent of the brain injury at the level of the four brain lobes (frontal, parietal, temporal, and occipital) of both cerebral hemispheres (right and left). The basal ganglia/brainstem score describes the involvement of subcortical structures (caudate, putamen, globus pallidus, thalamus, posterior limb of the internal capsule, brainstem) of both sides (right and left). The corpus callosum score assesses the involvement of this midline structure. Finally, the global score is the summary of the subscores plus a cerebellum score.^15^, ^16^ Brain lesions were also classified according to the MRICS into brain maldevelopments, predominant white matter injury, predominant grey matter injury, miscellaneous, or normal findings.^14^

Primary motor type, motor distribution, and GMFCS level were determined by two independent, trained physiotherapists.^23^ Eating and Drinking Abilities Classification System (EDACS)^24^ or Mini‐EDACS (≤3 years old)^25^ level was determined by a speech therapist based on clinical evaluation. Parents reported if a gastrostomy tube was used as the primary means of providing nutrition.

Height (196 occasions, 83 children) or supine length (124 occasions, 71 children) were measured to the last completed millimetre using a portable stadiometer or length‐measuring board (Shorr Productions LLC, Olney, MD, USA). Height was estimated from knee height (38 occasions, 30 children) measured using an anthropometer (Holtain Ltd., Crymych, UK).^26^ Weight was measured to the nearest 100 g using chair or portable electronic scales. Gestational age at birth (weeks) and birth weight (g) was reported by parents. Head circumference was measured with a flexible measuring tape (232 occasions, 109 children). Anthropometric data were converted to z‐scores using World Health Organization (<2 years) and Centers for Disease Control and Prevention (≥2 years) growth charts, as per standard clinical practice.^27^

In children aged 18 months to 5 years, total body water was determined using the deuterium dilution technique (146 occasions, 90 children); if unsuccessful, a validated bioelectrical impedance analysis method was used (123 occasions, 84 children).^28^ Children took a loading dose of deuterium in the form of water, orally, or via feeding tube. Urine samples were collected before dosing to determine natural baseline enrichment of the isotope, approximately 5 hours after dosing to calculate the body water pool using standard equations.^29^ Fat‐free mass was calculated using age‐specific and sex‐specific hydration constants^30^ and fat mass was determined. In 8‐ to 12‐year‐old children, a total body dual‐energy X‐ray absorptiometry scan (Lunar Prodigy, GE Healthcare, Madison, WI, USA) was used to obtain total body bone mineral content, lean total mass, and fat mass.^18^ Fat‐free mass was calculated by summing bone mineral content and lean total mass. The Fat‐Free Mass Index (FFMI) and Fat Mass Index (FMI) were calculated by normalizing fat‐free mass and fat mass for height (kg/m^2^).

### Statistical analysis

Descriptive data are reported as the mean and SD, or frequency and percentage. Differences in sqMRI scores between GMFCS groups were determined using a Welch's analysis of variance with Bonferroni correction. Differences between children with and without complete data were determined using Student's *t*‐tests and *χ* ^2^ tests. This was a secondary analysis of a population‐based cohort;^19^, ^20^ hence sample size calculations were not performed. Longitudinal analysis was undertaken via multilevel mixed‐effects regression models, which account for repeat measurements within participants. In all models, participants were entered as a random effect, while age and its quadratic term (age^2^) were included as fixed effects. Depending on the model, height (HTZ), weight (WTZ), or head circumference z‐score (HDZ), FFMI or FMI and age were included as repeated measures. The value of the *β* coefficient represents the average change in the outcome variable (growth or body composition measures) with a one‐unit change in the explanatory variable (sqMRI score). Directed acyclic graphs (DAGs) (Figures S2 and S3) were constructed using ‘daggity’ (v 3.1) to determine which confounders to include.^31^ The DAGs were constructed based on a literature review and clinical knowledge. To identify the total effect of the extent of the brain lesion on the outcomes (aim 1), gestational age at birth, birthweight, and sex were included as possible confounders. Because GMFCS level is on the causal path between the extent of the brain lesion and outcomes, including GMFCS as a variable would have led to bias through over‐adjustment.^32^ According to DAG modelling, adjusting only for sex and birthweight is sufficient; however, birthweight was only available for 68% of participants. Birthweight and gestational age at birth were highly correlated (*r* = 0.85); as the latter was available for all participants, it was used instead. In the secondary analyses, we wished to describe the growth and body composition of children with CP according to GMFCS level relative to typically developing children while adjusting for the extent of the brain lesion. In this instance, GMFCS is the exposure. According to the DAG, adjusting for the extent of the brain lesion, sex, and birthweight is sufficient, and we chose to only use an overall global score for simplicity and again substituted birthweight with gestational age at birth. Linear and quadratic terms for age at assessment (aims 1 and 2), gestational age at birth (aim 1), and interaction terms between GMFCS and age (aim 2), were evaluated. Age was centred at the youngest measured age (1 year 5 months) and gestational age at birth at the lowest gestational age (23 weeks) to avoid regressing to age or gestational age zero. After constructing the models, variables were retained in the final model if they were statistically significant at *p* < 0.05 using the likelihood ratio test. Normality of residuals was assessed using normality plots. Marginal effects at 2 years, 5 years, and 10 years were calculated to illustrate change over time, and gestational age at birth was set to term (40 weeks) for these analyses. Analyses were performed in Stata v13.0 (StataCorp, College Station, TX, USA).

## RESULTS

Parents/guardians of 176 eligible children consented (Figure S1). Of the 176 recruited children, 133 completed a brain MRI; of these, 124 children had complete growth and feeding mode data from 358 assessment points and 118 children had body composition data from 307 assessment points. Children were seen for an average of 2.1 (1.0) assessments, and the mean age at brain MRI was 2 years 1 month (1 years 11 months); 44% were aged 2 years or older at the MRI assessment. Participant characteristics are reported according to GMFCS level at age 5 years in Table 1 and according to age group in Table S1. Participants with missing data did not differ from participants included in the study (Table S2). Study participants did not markedly differ from the Australian population of 5‐year‐olds with CP regarding GMFCS level or motor type (Table S2).

**TABLE 1:** Participant characteristics according to Gross Motor Function Classification System at 5 years old.

	GMFCS level I, *n* = 50	GMFCS level II, *n* = 24	GMFCS level III, *n* = 17	GMFCS level IV, *n* = 12	GMFCS level V, *n* = 21
Age, years:months	4:10 (0:10)	5:2 (0:4)	5:0 (0:8)	4:9 (1:0)	4:7 (1:1)
Gestational age at birth, weeks	36.60 (4.15)	34.42 (5.63)	33.53 (5.58)	36.50 (5.27)	36.71 (4.67)
Birthweight, g	2802 (949)	2283 (954)	2142 (1110)	2450 (927)	2698 (1138)
Height z‐score	0.20 (1.20)	−0.51 (0.90)	−1.33 (1.17)	−1.32 (1.69)	−1.01 (1.39)
Weight z‐score	0.19 (1.09)	−0.63 (1.38)	−1.18 (1.39)	−0.84 (2.08)	−0.97 (1.96)
Head circumference z‐score (*n* = 82)	0.40 (1.19) (*n* = 35)	−0.31 (0.90) (*n* = 16)	−0.81 (1.44) (*n* = 14)	−1.39 (2.20) (*n* = 6)	−1.30 (2.32) (*n* = 11)
Fat‐Free Mass Index	12.29 (1.20)	11.77 (1.44)	11.77 (1.29)	12.40 (1.43)	11.72 (1.62)
Fat Mass Index	3.25 (1.26)	3.52 (0.94)	3.37 (0.69)	4.36 (1.55)	4.55 (1.43)
Semi‐quantitative magnetic resonance imaging brain lesion scores^a^ (significantly different from the GMFCS level I group)					
Hemispheric	6.31 (4.03)	8.35 (4.03)	10.00 (3.87)^a^ *p* < 0.001	10.33 (5.06)^a^ *p* = 0.001	10.29 (8.39)^a^ *p* = 0.001
Sum of basal ganglia/brainstem	2.44 (3.54)	1.96 (3.79)	1.41 (2.45)	2.92 (4.44)	5.62 (7.31)
Corpus callosum	1.02 (1.10)	1.21 (1.38)	1.29 (1.36)	2.58 (2.43)	1.90 (1.34)
Global score total (0–48)	9.77 (7.00)	11.94 (7.18)	12.94 (5.19)	15.92 (9.25)	18.38 (15.73)
Sex, male, *n* (%)	32 (64)	16 (67)	12 (71)	6 (50)	11 (52)
Magnetic Resonance Imaging Classification System, *n* (%)					
Predominant grey matter injury	11 (22)	1 (4)	1 (5)	2 (17)	9 (43)
Predominant white matter injury	31 (62)	17 (71)	13 (76)	8 (67)	6 (29)
Brain maldevelopment	2 (4)	2 (8)	0 (0)	1 (8)	1 (5)
Miscellaneous	2 (4)	2 (8)	3 (18)	1 (8)	2 (10)
Normal magnetic resonance imaging	4 (8)	2 (8)	0 (0)	0 (0)	3 (14)
Primary motor type, *n* (%)					
Unilateral spasticity	32 (64)	6 (25)	1 (6)	0 (0)	0 (0)
Bilateral spasticity	15 (30)	14 (58)	13 (77)	10 (83)	13 (62)
Dystonic, ataxic, hypotonic, dyskinetic	3 (6)	4 (17)	3 (18)	2 (17)	8 (38)
EDACS level, *n* (%)					
I	44 (90)	13 (54)	3 (18)	2 (18)	0 (0)
II	2 (5)	10 (42)	9 (53)	2 (18)	0 (0)
III	1 (2.5)	0 (0)	5 (29)	2 (18)	2 (10)
IV	0 (0)	1 (4)	0 (0)	3 (27)	3 (15)
V	0 (0)	0 (0)	0 (0)	2 (18)	15 (75)
Missing	1 (2.5)	–	–	–	–
Primary feeding mode, tube‐fed, *n* (%)	0 (0)	0 (0)	1 (6)	1 (8)	16 (76)

*Note*: Data are mean (SD) unless otherwise indicated.

Abbreviations: EDACS, Eating and Drinking Abilities Classification System; GMFCS, Gross Motor Function Classification System.

^a^ Welch's analysis of variance for differences between groups with Bonferroni correction for multiple comparisons.

### Height z‐score

For aim 1, in the final model, age, gestational age at birth, and its quadratic term were retained. Gestational age at birth was positively associated with HTZ; however, the quadratic term was negative and significantly added to the model, indicating that the effect of gestational age at birth was negatively curvilinear, that is, largest for infants born very preterm (23 weeks). Then, the effect of gestational age at birth on HTZ grew progressively smaller for each week closer to term a child was born. Global, basal ganglia/brainstem, corpus callosum, and hemispheric scores were significantly and inversely associated with HTZ (Table 2).

**TABLE 2:** Associations between semi‐quantitative MRI scores (extent of brain lesion), growth, and body composition in children with cerebral palsy.

Score	Height z‐score^a^ (*n* = 124)	Weight z‐score^a^ (*n* = 124)	Head circumference z‐score^b^ (*n* = 109)	Fat‐Free Mass Index^c^ (*n* = 118)	Fat Mass Index^c^ (*n* = 118)
	*β* (95% CI)	*β* (95% CI)	*β* (95% CI)	*β* (95% CI)	*β* (95% CI)
Unadjusted global score	−0.05 (−0.07 to −0.03)***, *p* < 0.001	−0.03 (−0.06 to −0.01)**, *p* = 0.010	−0.03 (−0.07 to 0), *p* = 0.062	−0.005 (−0.03 to 0.02), *p* = 0.674	−0.01 (−0.03 to 0.02), *p* = 0.612
Adjusted global score	−0.04 (−0.07 to −0.02)***, *p* < 0.001	−0.03 (−0.06 to −0.004)*, *p* = 0.020	−0.03 (−0.07 to 0.001), *p* = 0.053	−0.01 (−0.03 to 0.01), *p* = 0.522	−0.004 (−0.03 to 0.02), *p* = 0.733
Unadjusted basal ganglia/brainstem	−0.06 (−0.11 to −0.01)*, *p* = 0.017	−0.05 (−0.11 to 0.004), *p* = 0.070	−0.04 (−0.11 to 0.04), *p* = 0.325	0.004 (−0.04 to 0.05), *p* = 0.860	−0.01 (−0.06 to 0.04), *p* = 0.706
Adjusted basal ganglia/brainstem	−0.06 (−0.11 to −0.02)**, *p* = 0.009	−0.06 (−0.11 to −0.004)*, *p* = 0.035	−0.04 (−0.11 to 0.03), *p* = 0.293	0.002 (−0.04 to 0.05), *p* = 0.922	−0.02 (−0.06 to 0.03), *p* = 0.473
Unadjusted corpus callosum	−0.27 (−0.41 to −0.12)***, *p* < 0.001	−0.23 (−0.40 to −0.06)*, *p* = 0.008	−0.14 (−0.36 to 0.07), *p* = 0.197	−0.11 (−0.25 to 0.04), *p* = 0.152	−0.01 (−0.18 to 0.15), *p* = 0.940
Adjusted corpus callosum	−0.20 (−0.36 to −0.05)**, *p* = 0.009	−0.14 (−0.31 to 0.04), *p* = 0.130	−0.14 (−0.37 to 0.7), *p* = 0.180	−0.09 (−0.24 to 0.05), *p* = 0.205	−0.03 (−0.18 to 0.13), *p* = 0.744
Unadjusted hemispheric	−0.09 (−0.13 to −0.05)**, *p* < 0.001	−0.05 (−0.10 to −0.003)*, *p* = 0.048	−0.06 (−0.12 to 0), *p* = 0.050	−0.0004 (−0.04 to 0.03), *p* = 0.802	−0.01 (−0.06 to 0.03), *p* = 0.552
Adjusted hemispheric	−0.08 (−0.12 to −0.04)*** *p* < 0.001	−0.04 (−0.09 to 0.004), *p* = 0.075	−0.06 (−0.119 to −0.001)*, *p* = 0.046	−0.01 (−0.05 to 0.03), *p* = 0.542	0.0003 (−0.04 to 0.04), *p* = 0.988

*Note*: Mixed linear regression.

Abbreviations: CI, confidence interval; MRI, magnetic resonance imaging.

^a^ Adjusted for age, gestational age at birth, and gestational age at birth^2^.

^b^ Adjusted for age and age^2^.

^c^ Adjusted for age, age^2^, and sex.

* *p* ≤ 0.05.

** *p* ≤ 0.01.

*** *p* ≤ 0.001.

**FIGURE 1:**
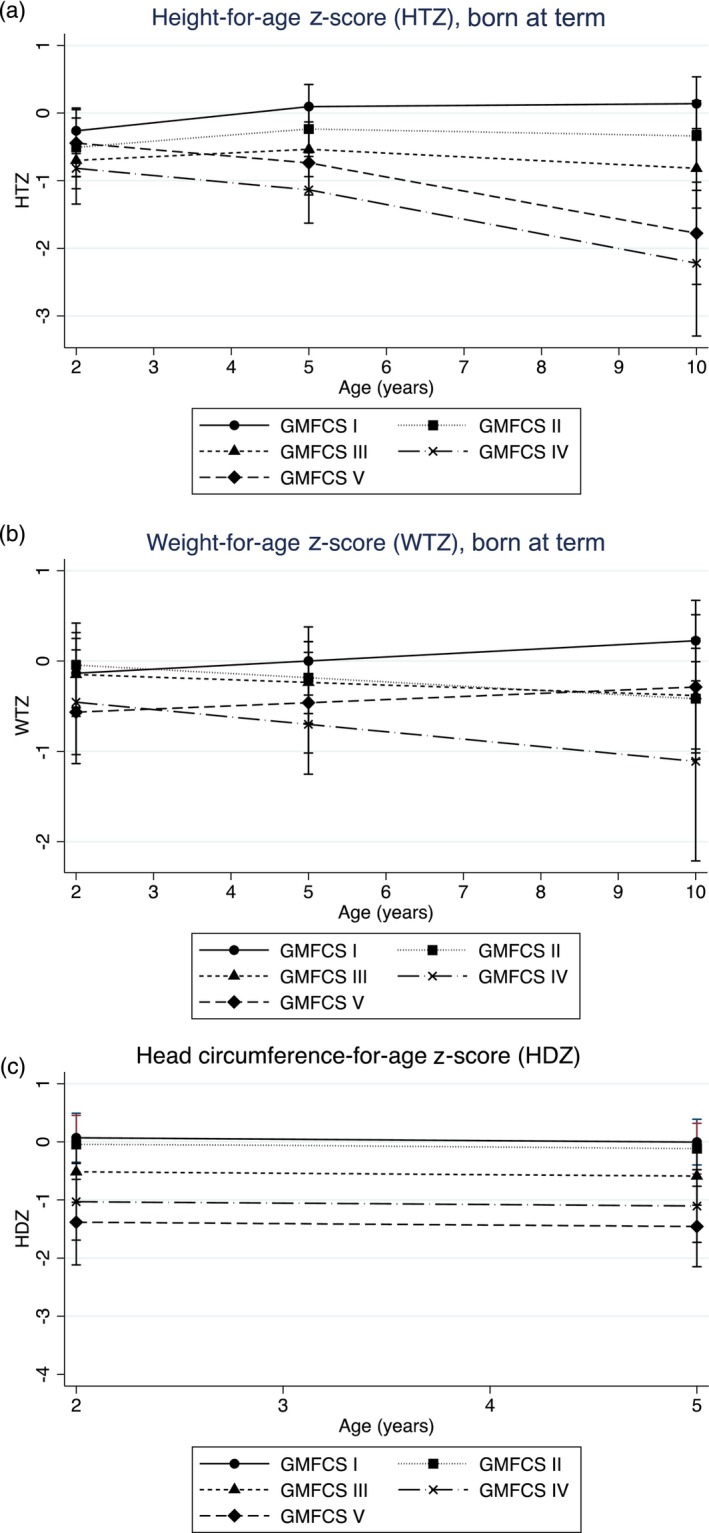
HTZ (a) and WTZ (b) z‐scores for children born at term, and HDZ (c) aged from 2 to 10 years according to GMFCS group (*n* = 124). Abbreviations: GMFCS, Gross Motor Function Classification System; HDZ, head circumference z‐score; HTZ, height z‐score; WTZ, weight z‐score.

For aim 2, age and gestational age at birth, their quadratic terms, an interaction term for GMFCS and age, and global score were included (Table 3). There was no significant difference in HTZ between the GMFCS level I, II, III, and V groups at baseline; the GMFCS level IV group was significantly shorter than the GMFCS level I group (Table 3). The GMFCS level I group had a significant increase in HTZ with age. While the GMFCS level II group did not differ from the GMFCS level I group, the GMFCS level III and IV groups showed a non‐significant slower trajectory with age relative to the GMFCS level I group, and the GMFCS level V group grew significantly slower with age. Overall, the association with age was negatively curvilinear. The association with gestational age at birth remained as described earlier. At 2 years old, children classified in GMFCS levels II, III, and IV were significantly shorter than the reference children (Table 4 and Figure 1a). At 5 years old and 10 years old, children classified in GMFCS levels III, IV, and V were significantly shorter than the reference children.

**TABLE 3:** Growth and body composition in children with cerebral palsy by Gross Motor Function Classification System.

	Height z‐score *n* = 124	Weight z‐score *n* = 124	Head circumference z‐score *n* = 109	Fat‐Free Mass Index *n* = 118	Fat Mass Index *n* = 118
	*β* (95% CI)	*β* (95% CI)	*β* (95% CI)	*β* (95% CI)	*β* (95% CI)
GMFCS level					
I	Ref	Ref	Ref	Ref	Ref
II	−0.25 (−0.64 to 0.14)	−0.03 (−0.31 to 0.24)	−0.11 (−0.53 to 0.31)	−0.53 (−0.98 to −0.09)*	1.00 (0.07 to 1.95)*
III	−0.42 (−0.90 to 0.05)	−0.19 (−0.60 to 0.21)	−0.60 (−1.16 to −0.03)*	−0.56 (−1.10 to −0.02)*	0.51 (−0.43 to 1.46)
IV	−0.64 (−1.28 to −0.002)*	−0.60 (−1.12 to −0.10)*	−1.12 (−1.82 to −0.42)**	−0.75 (−1.39 to −0.11)*	1.56 (0.20 to 2.92)*
V	−0.65 (−0.65 to 0.54)	−0.47 (−1.06 to 0.12)	−1.46 (−2.25 to −0.67)***	−1.22 (−1.80 to −0.64)***	0.23 (−0.78 to 1.24)
Age^a^	n/a	n/a	−0.19 (−0.59 to 0.21)	−0.78 (−1.02 to −0.55)***	n/a
GMFCS level I × age (Ref)	0.39 (0.13 to 0.66)**	0.29 (0.07 to 0.52)	n/a	n/a	−0.03 (−0.30 to 0.24)
GMFCS level II × age	0 (−0.16 to 0.16)	−0.06 (−0.22 to 0.09)	n/a	n/a	−0.10 (−0.27 to 0.06)
GMFCS level III × age	−0.09 (−0.23 to 0.06)	−0.01 (−0.16 to 0.14)	n/a	n/a	−0.07 (−0.25 to 0.11)
GMFCS level IV × age	−0.18 (−0.38 to 0.02)	−0.04 (−0.24 to 0.16)	n/a	n/a	−0.19 (−0.50 to 0.12)
GMFCS level V × age	−0.26 (−0.42 to −0.10)***	0.12 (−0.03 to 0.28)	n/a	n/a	0.29 (0.09 to 0.50)**
Age^2^	−0.06 (−0.12 to −0.01)**	n/a	0.04 (−0.05 to 0.13)	0.05 (0.03 to 0.07)***	−0.03 (0.01 to 0.05)**
Gestational age at birth	0.21 (0.03 to 0.40)*	0.29 (0.07 to 0.52)*	n/a	n/a	n/a
Gestational age at birth^2^	−0.01 (0.02 to 0)	−0.01 (−0.02 to 0.003)	n/a	n/a	n/a
Sex	n/a	n/a	n/a	n/a	0.54 (0.13 to 0.94)**
Global	−0.03 (−0.05 to −0.01)**	−0.02 (−0.05 to 0.003)	−0.01 (−0.05 to 0.02)	0.01 (−0.01 to 0.03)	−0.02 (−0.04 to 0)

Abbreviations: CI, confidence interval; GMFCS, Gross Motor Function Classification System; Ref, reference group.

^a^ Centred at 18 months.

* *p* ≤ 0.05.

** *p* ≤ 0.01.

*** *p* ≤ 0.001.

**TABLE 4:** Predicted growth and body composition at 2 years and 5 years of age for children with cerebral palsy according to Gross Motor Function Classification System level relative to typically developing children.

		2 years^a^		5 years		10 years	
		Margin (95% CI)		Margin (95% CI)		Margin (95% CI)	
HTZ 2 years: *n* = 33 5 years: *n* = 100 10 years: *n* = 38	GMFCS level I	−0.26 (−0.59 to 0.07)		0.09 (−0.23 to 0.42)		0.13 (−0.26 to 0.54)	
	GMFCS level II	−0.51 (−0.94 to −0.07)*		−0.23 (−0.59 to 0.11)		−0.34 (−0.86 to 0.18)	
	GMFCS level III	−0.70 (−1.11 to −0.28)***		−0.53 (−0.94 to −0.13)***		−0.82 (−1.41 to −0.23)***	
	GMFCS level IV	−0.82 (−1.35 to −0.28)**		−1.14 (−1.63 to −0.64)**		−2.22 (−3.29 to −1.14)***	
	GMFCS level V	−0.44 (−0.94 to 0.05)		−0.73 (−1.21 to −0.26)**		−1.78 (−2.53 to −1.02)**	
WTZ 2 years: *n* = 33 5 years: *n* = 100 10 years: *n* = 38	GMFCS level I	−0.13 (−0.52 to 0.25)		0 (−0.37 to 0.37)		0.23 (−0.22 to 0.67)	
	GMFCS level II	−0.04 (−0.51 to 0.42)		−0.18 (−0.58 to 0.21)		−0.42 (−0.97 to 0.14)	
	GMFCS level III	−0.15 (−0.61 to 0.31)		−0.24 (−0.69 to 0.22)		−0.38 (−1.12 to 0.25)	
	GMFCS level IV	−0.46 (−1.03 to 0.12)		−0.70 (−1.25 to −0.15)*		−1.11 (−2.21 to −0.01)*	
	GMFCS level V	−0.57 (−1.14 to 0)		−0.46 (−1.02 to 0.10)		−0.29 (−1.09 to 0.51)	
HDZ 2 years: *n* = 33 5 years: *n* = 82	GMFCS level I	0.07 (−0.35 to 0.49)		−0 (−0.40 to 0.39)		n/a	
	GMFCS level II	−0.04 (−0.54 to 0.46)		−0.12 (−0.55 to 0.32)		n/a	
	GMFCS level III	−0.51 (−1.04 to 0.01)		−0.59 (−1.09 to −0.09)*		n/a	
	GMFCS level IV	−1.03 (−1.69 to −0.37)**		−1.10 (−1.73 to −0.48)***		n/a	
	GMFCS level V	−1.38 (−2.12 to 0.65)***		−1.46 (−2.15 to −0.76)***		n/a	
		Males (*n* = 19)	Females (*n* = 12)	Males (*n* = 56)	Females (*n* = 32)	Males (*n* = 24)	Females (*n* = 12)
		Margin (95% CI)	Margin (95% CI)	Margin (95% CI)	Margin (95% CI)	Margin (95% CI)	Margin (95% CI)
Fat‐Free Mass Index	Typically developing, mean (SD)^b^	12.87 (1.15)	12.34 (1.18)	13.00 (1.14)	12.17 (1.29)	13.75 (1.41)	13.20 (1.62)
	GMFCS level I	13.68 (13.30 to 14.06)	13.49 (13.06 to 13.93)	12.37 (12.03 to 12.70)	12.18 (11.78 to 12.58)	12.16 (11.69 to 12.62)	11.97 (11.45 to 11.53)
	GMFCS level II	13.15 (12.66 to 13.63)	12.96 (12.42 to 13.50)	11.84 (11.41 to 12.17)	11.65 (11.17 to 12.14)	11.62 (11.09 to 12.16)	11.44 (10.86 to 12.02)
	GMFCS level III	13.12 (12.61 to 13.63)	12.93 (12.35 to 13.51)	11.81 (11.31 to 12.30)	11.62 (11.05 to 12.19)	11.60 (11.00 to 12.19)	11.41 (10.75 to 12.07)
	GMFCS level IV	12.94 (12.31 to 13.57)	12.75 (12.10 to 13.40)	11.63 (11.03 to 12.23)	11.44 (10.82 to 12.07)	11.42 (10.74 to 12.12)	11.23 (10.51 to 11.96)
	GMFCS level V	12.48 (11.91 to 13.05)	12.29 (11.73 to 12.86)	11.17 (10.63 to 11.71)	10.98 (10.44 to 12.49)	10.96 (13.31 to 11.60)	10.77 (10.12 to 11.42)
Fat Mass Index	Typically developing, mean (SD)^b^	3.82 (1.40)	3.90 (1.40)	3.67 (1.48)	3.25 (1.42)	3.60 (1.73)	4.22 (1.55)
	GMFCS level I	2.65 (2.23 to 3.07)	3.17 (2.69 to 3.64)	3.18 (2.84 to 3.52)	3.70 (3.28 to 4.11)	5.23 (4.67 to 5.77)	5.75 (5.14 to 6.35)
	GMFCS level II	3.45 (2.79 to 4.11)	3.97 (3.27 to 4.66)	3.67 (3.23 to 4.12)	4.19 (3.69 to 4.69)	5.21 (4.34 to 6.08)	5.73 (4.83 to 6.63)
	GMFCS level III	3.01 (2.38 to 3.65)	3.53 (2.83 to 4.23)	3.32 (2.79 to 3.85)	3.83 (3.22 to 4.44)	4.99 (3.90 to 6.08)	5.51 (4.39 to 6.63)
	GMFCS level IV	3.83 (3.01 to 4.65)	4.35 (3.52 to 5.17)	3.79 (3.09 to 4.48)	4.30 (3.59 to 5.02)	4.88 (2.90 to 6.85)	5.40 (3.41 to 7.39)
	GMFCS level V	3.47 (2.81 to 4.13)	3.98 (3.33 to 4.64)	4.87 (4.31 to 5.44)	5.39 (4.83 to 5.96)	8.39 (7.12 to 9.66)	8.90 (7.63 to 10.18)

Abbreviations: CI, confidence interval; GMFCS, Gross Motor Function Classification System, HDZ, head circumference z‐score; HTZ, height z‐score; WTZ, weight z‐score.

^a^ 2 years chosen for body composition because the available reference data start at 2 years of age for body composition. Mixed linear regression, significantly different from z‐score = 0, that is, age‐matched and sex‐matched 50th centile for typically developing children using Centers for Disease Control and Prevention growth charts (*p ≤ 0.05, **p ≤ 0.01, ***p ≤ 0.001).

^b^ Fat Mass Index and Fat‐Free Mass Index references in children and young adults: assessments along racial and ethnic lines.^33^

### Weight z‐score

For aim 1, in the final model, age, gestational age at birth, and its quadratic term were retained. As with HTZ, the association with gestational age at birth was positive but negatively curvilinear as for HTZ. Global and basal ganglia/brainstem scores had significant inverse associations with WTZ; corpus callosum and hemispheric scores were not associated with WTZ (Table 2).

For aim 2, age, gestational age at birth and its quadratic term, and global score were included. The GMFCS level V group was significantly lighter than the GMFCS level I group at baseline and WTZ; there was a non‐significant increase in WTZ over time for children in the GMFCS level I group. The change in WTZ over time showed a non‐significant slower trajectory for the GMFCS level II, III, and IV groups, and a non‐significant faster trajectory for the GMFCS level V group (Table 3). There was a significant positive association between gestational age at birth and WTZ. The global brain lesion score was no longer significant after including GMFCS in the model. At 2 years, all GMFCS groups had a similar weight to the reference group, but by age 5 years and 10 years, children in the GMFCS level IV group were significantly lighter than the children in the reference group (Table 4 and Figure 1b).

### Head circumference z‐score

For aim 1, age and its quadratic term were retained in the final model. The hemispheric score was significantly associated with smaller HDZ (*β* = −0.06, 95% confidence interval [CI] = −0.11 to −0.001). Global, basal ganglia/brainstem, and corpus callosum scores were not associated with HDZ.

For aim 2, age, its quadratic term, and GMFCS level were retained in the final model. The GMFCS level II, III, IV, and V groups had significantly smaller HDZ at baseline relative to the GMFCS level I group, and there was a non‐significant decrease in HDZ with age. At 2 years, children in the GMFCS level IV and V groups had significantly smaller HDZ than typically developing children in the reference group. By age 5 years, children in the GMFCS level III, IV, and V groups had significantly smaller HDZ (Table 4 and Figure 1c). Head circumference was only available up to 5 years of age.

### Fat‐Free Mass Index

For aim 1, in the final model, age and its quadratic term were retained. Age was negatively associated with FFMI but had a positive curvilinear association (i.e. the decrease in FFMI with age slowed down with increasing age) with FFMI. None of the sqMRI scores were significantly associated with FFMI (Table 2).

**FIGURE 2:**
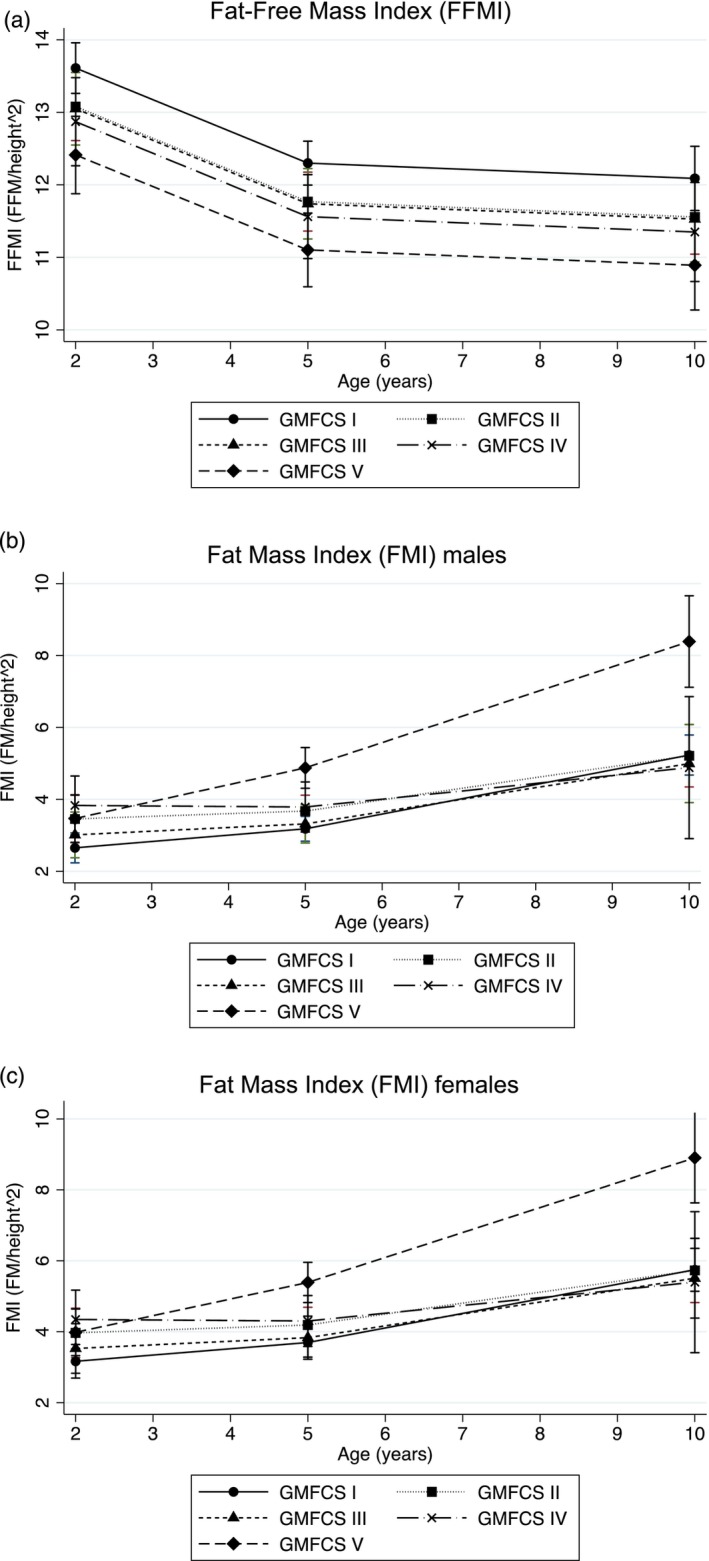
FFMI (a) and FMI for males (b) and females (c) aged from 2 to 10 years according to GMFCS group (*n* = 114). Abbreviations: FFMI, Fat‐Free Mass Index; FMI, Fat Mass Index; GMFCS, Gross Motor Function Classification System.

For aim 2, age and its quadratic term, GMFCS, and global score were included. The GMFCS level II, III, IV, and V groups had significantly lower FFMI than the GMFCS level I group. FFMI decreased with age; however, the decrease was greater during the younger ages and slowed down with time (i.e. it was positively curvilinear) (Figure 2a). Table 4 shows the predicted FFMI at ages 2 years, 5 years, and 10 years for each GMFCS group and sex relative to the reference data.

### Fat Mass Index

For aim 1, in the final model, age and its quadratic term, and sex were retained. The association with age was positive and positively curvilinear (i.e. the association between age and FMI increased with age). Females had a significantly greater FMI than males. None of the sqMRI scores were significantly associated with FMI (Table 2).

For aim 2, age, quadratic age, an interaction term for GMFCS and age, sex, and global score were included. Children in the GMFCS level II and IV groups had significantly higher FMI relative to children in the GMFCS level I group. Over time, FMI significantly increased for children in the GMFCS level II and V groups. While initially there was no statistically significant change with age for the GMFCS level I, II, III, or IV groups (Table 3 and Figure 2b,c), the association with age was positively curvilinear (i.e. it increased with age). Females had a significantly greater FMI than males. Table 4 shows the predicted FMI at ages 2 years, 5 years, and 10 years for each GMFCS group and sex relative to the reference data.

### MRI classification system

There were no significant differences in FFMI or FMI, HDZ, HTZ, or WTZ between MRICS groups (Table S3).

## DISCUSSION

In a longitudinal, population‐based sample of children with CP aged 18 months to 13 years, a greater extent of the brain lesion, as determined using an sqMRI scoring method, was significantly associated with shorter stature, lower body weight, and smaller head circumference relative to age‐matched and sex‐matched typically developing children (reference population) after adjusting for gestational age at birth. The global score and all subscores (basal ganglia/brainstem, corpus callosum, and hemispheric scores) were significantly associated with HTZ; global and basal ganglia/brainstem scores were associated with WTZ and hemispheric scores were associated with HDZ. There were no associations between the extent of the brain lesion and body composition. This suggests that there are significant neurological impacts on growth in every dimension for children with CP. We still need to elucidate the specific mediator pathways responsible for these changes (e.g. hormones, metabolism, dysphagia). The specific subscores significantly associated with the outcomes may give an indication of the mechanisms involved but larger samples across GMFCS levels using more sophisticated MRI technology are required.

The secondary aim of this study was to describe longitudinal growth and body composition in children with CP aged between 18 months and 13 years according to GMFCS level after adjusting for the extent of the brain lesion. Adjusting for the negative association between the extent of the brain lesion and growth, children in the GMFCS level II, III, and IV groups were significantly shorter than the reference children at age 2 years (Table 4 and Figure 1a). However, by ages 5 years and 10 years, only children classified in GMFCS levels III, IV, and V were significantly shorter than the reference children. This probably demonstrates the impact of ambulatory status on linear growth. At the age of 2 years, there were no differences in WTZ for any GMFCS group relative to the reference population; however, by ages 5 years and 10 years, children in the GMFCS level IV group were significantly lighter (Table 4 and Figure 1b). These findings for weight are likely (at least in part) due to the independent effects of feeding difficulty and feeding mode on growth. Most children in the GMFCS level IV (*n* = 7 of 12) and V (*n* = 21 of 21) groups were classified as EDACS levels III to V (Table 1). This indicates limitations in efficiency and safety in eating and drinking,^24^ which can compromise nutritional adequacy and growth in every dimension. However, children classified in GMFCS level V were primarily tube‐fed (*n* = 16 of 21), while children classified in GMFCS level IV (*n* = 11 of 12) were primarily fed orally. Indeed, US growth charts for children with CP demonstrate that children classified in GMFCS level V who are tube‐fed follow a higher height and weight trajectory relative to orally fed children classified in GMFCS level V,^4^ whereas two longitudinal cohort studies identified that children with CP who had feeding difficulties in infancy^2^ or a dysphagia diagnosis^3^ had significantly lower HTZ and WTZ. Because of the strong correlation between GMFCS and EDACS (*r* = 0.86), these variables could not be included in the same model. Small sample sizes for individual EDACS levels and tube‐fed versus orally fed children limited our ability to explore these effects in our sample in separate analyses. Gestational age at birth also has a significant impact on HTZ and WTZ, with the largest effect seen for those born very preterm. Other studies have used a dichotomous variable for preterm birth status and found no difference in growth between groups;^3^ however, this does not take into account the differences between children born extremely versus late preterm. Furthermore, in contrast to the findings for HTZ, the association between global sqMRI score and WTZ was attenuated after including GMFCS level in the model, indicating that a large portion of the effect of brain lesion scores on WTZ is mediated through the level of gross motor impairment; mediation analyses in larger samples would allow us to explore this.

There was no association between the extent of the brain lesion and body composition; however, significant differences in body composition were observed depending on GMFCS level (Figure 2a–c). Between the ages of 2 years and 10 years, typically developing children demonstrate a slight increase in FFMI and a decrease in FMI (Table 4).^33^ However, in the study population, all GMFCS groups displayed a significant reduction in FFMI over time (Table 3 and Figure 2a); while children in the GMFCS level I group had faster linear growth, gains in fat‐free mass did not match long bone growth, which is consistent with previous studies.^34^ The GMFCS level V group had a significant increase in FMI over time (Table 3 and Figure 2b,c). Furthermore, quadratic age demonstrated an overall negative curvilinear association with FFMI over time, and a positive curvilinear association with FMI over time. Observed on its own, the finding that children classified in GMFCS level V did not differ in weight from reference children by age 10 years may be seen as positive; however, their body composition measures demonstrate that this is due to excessive fat mass gain, which may be iatrogenic and preventable with careful monitoring. The pattern in body composition change over time for all GMFCS levels has detrimental long‐term consequences for metabolic health for individuals with CP.^35^

Findings on fat mass in children with CP vary;^9^, ^10^ however, fat‐free mass is consistently reported to be lower in terms of both bone density and muscle mass relative to typically developing (reference) children.^6^, ^7^, ^8^ The inconsistent findings for fat mass may stem from regional differences in medical treatment and feeding support. Fat‐free mass would also be affected by inadequate nutrition; however, in the current sample, FFMI developed abnormally despite normalized weight and height status for children in the GMFCS level I group by 5 years of age. Indeed, differences in muscle volume that persist longitudinally with increasing disparities with age for children classified in GMFCS levels I and II, and III and IV, have been observed from 12 months of age in children with CP relative to typically developing children.^36^ Abnormal muscle growth could be due to altered neural input, motor disability, nutrition, growth factors, and metabolic capacity.^36^ Previous analyses of the current cohort found that greater habitual physical activity and energy intake were positively associated with FFMI.^22^ Further research is needed to understand the drivers of altered development of bone and muscle in children with CP.

Despite the study's strengths, such as a longitudinal, population‐based sample comparable to the Australian population with CP, limitations like sample size must be considered, particularly when examining the GMFCS level III and IV groups, because CIs are wide, and when extrapolating findings to the final time point because of loss to follow‐up. The absence of MRI data for 51 children reduced the power to detect differences. The cohort had predominantly white matter injury (60%), which limited the exploration of differences between brain lesion types. Just over half of the children (56%) had an MRI completed before 2 years of age and may not have completed the myelination process. Findings may only be generalizable to high‐income countries with universal health care like Australia and are specific to the brain lesions of children with a CP diagnosis.

In conclusion, in children with CP, differences in the extent and location of the brain lesion measured on an sqMRI scale were associated with linear growth, body weight, and head circumference, but not body composition. This suggests there are significant non‐nutritional impacts on growth in every dimension for children with CP. Furthermore, after adjusting for the overall extent of the brain lesion, GMFCS level was associated with differences in both growth and body composition. While children classified in GMFCS levels I and II had height, weight, and head circumference like those of typically developing (reference) children by ages 5 years and 10 years, their body composition trajectory was non‐typical. Children classified in GMFCS levels III to V were on average shorter than reference children, and all groups demonstrated non‐typical body composition trajectories. The complex interplay of nutritional and non‐nutritional factors complicates determining the optimal growth for children with CP. A better understanding of how the extent and location of the brain lesion affects growth may assist in interpreting a child's growth trajectory; however, further work is needed. It is also crucial to conduct further research that allow us to assess if early nutrition and physical activity interventions can alter the growth and body composition trajectories in children with CP and determine the impacts on long‐term health and well‐being.

## Supporting information


**Figure S1:** Flow chart of participant inclusion/exclusion.

**Figure S2:** Causal DAGs.

**Figure S3:** Directed Acyclic Graph for Aim 2.

**Table S1:** Participant characteristics by age group

**Table S2:** Comparison of included sample to excluded due to missing data and Australian CP register

**Table S3:** Prediction of height and weight z‐score, fat‐free mass and Fat Mass Index by brain lesion MRICS

## Data Availability

Data available on reasonable request from the authors.
