# Supplementary figures and images for: Brain lesion extent, growth, and body composition in children with cerebral palsy
Source: Dev Med Child Neurol. 2025 Jul 31;68(2):199–210. doi: 10.1111/dmcn.16427 (PMC12766548; doi:10.1111/dmcn.16427)

**Supplementary Figure 1: Flow chart of participant inclusion/ exclusion**

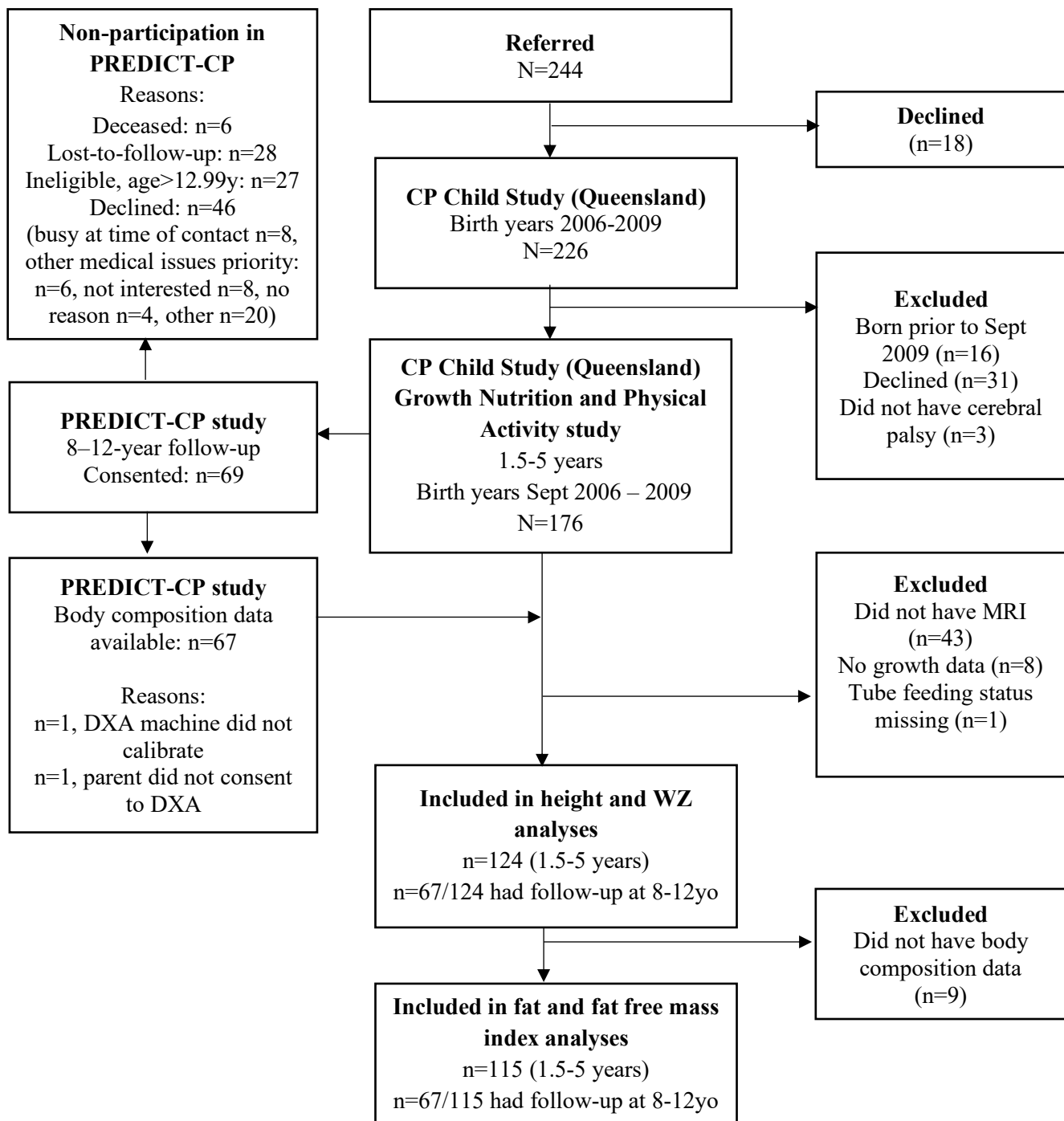

Supplement: Supplementary file 1 — Figure S1: Flow chart of participant inclusion/exclusion. [file DMCN-68-199-s006.pdf]
