# Supplementary material for: Brain lesion extent, growth, and body composition in children with cerebral palsy
Source: Dev Med Child Neurol. 2025 Jul 31;68(2):199–210. doi: 10.1111/dmcn.16427 (PMC12766548; doi:10.1111/dmcn.16427)
Supplement: Supplementary file 2 — Figure S2: Causal DAGs. [file DMCN-68-199-s001.pdf]

Supplemental Figures 2(a) and (b) Directed Acyclic Graphs for Aim 1: Total effect of extent of brain dysplasia on longitudinal (a) height/weight/ head circumference Z-score and (b) fat free and fat mass index in children with CP aged 1.5-13.0 years. Note, these are not intended to be exhaustive.

For (a) the only open biasing paths are through open biasing paths are through birth weight and sex. Gestational age used as substitute for birth weight to maximise sample size as they were highly correlated (r=0.85, p<0.001). Sex was only included in the fat mass index analyses and gestational age at birth to height and weight Z-score analyses as they did not significantly add to other models.

Legend: Green (exposure), Blue with I (outcome), Pink (ancestor of exposure and outcome, i.e., confounder), Blue (ancestor of outcome), Grey (unobserved/ latent)

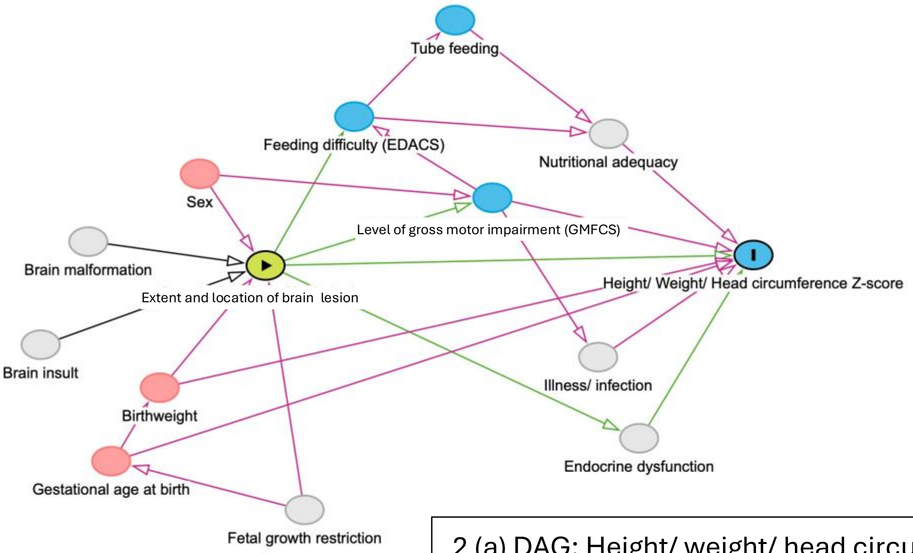

2 (a) DAG: Height/ weight/ head circumference z-

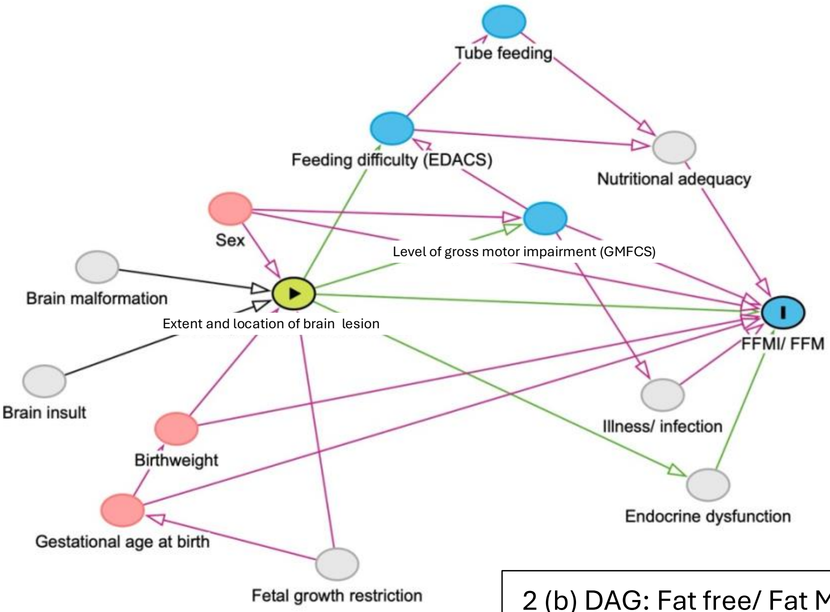

2 (b) DAG: Fat free/ Fat Mass Index
