# Supplementary material for: Brain lesion extent, growth, and body composition in children with cerebral palsy
Source: Dev Med Child Neurol. 2025 Jul 31;68(2):199–210. doi: 10.1111/dmcn.16427 (PMC12766548; doi:10.1111/dmcn.16427)
Supplement: Supplementary file 3 — Figure S3: Directed Acyclic Graph for Aim 2. [file DMCN-68-199-s004.pdf]

Supplemental Figures 3(a) and (b) Directed Acyclic Graph for Aim 2: Describe longitudinal (a) height/weight/ head circumference Z-score and (b) fat free and fat mass index by GMFCS in children with CP aged 1.5-13.0 years relative to typically developing children. Note, these are not intended to be exhaustive.

For (a) the only open biasing paths are through open biasing paths are through birth weight and sex. Sex was only included in the fat mass index analyses and gestational age at birth to height and weight Z-score analyses as they did not significantly add to other models.

Legend: Green (exposure), Blue(I) (outcome), Pink (ancestor of exposure and outcome, i.e., confounder), Blue (ancestor of outcome), Grey (unobserved/ latent)

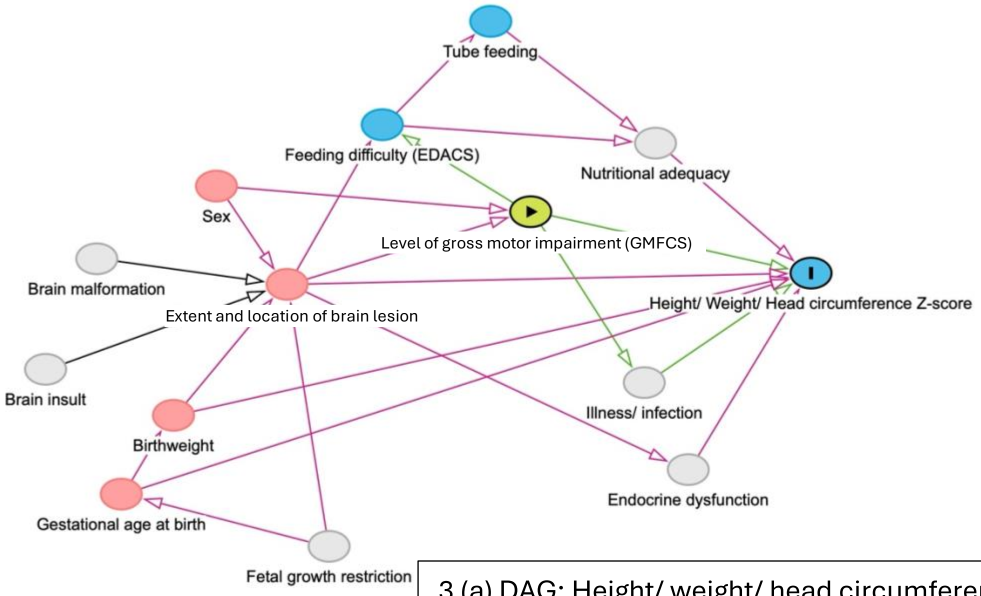

3 (a) DAG: Height/ weight/ head circumference z-

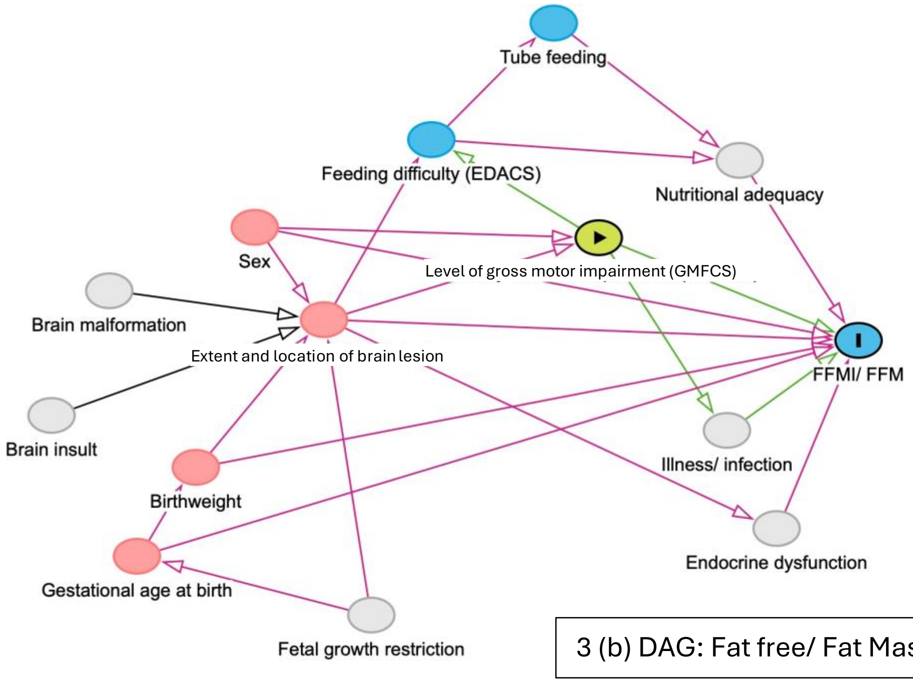

3 (b) DAG: Fat free/ Fat Mass Index
