# Supplementary material for: Brain lesion extent, growth, and body composition in children with cerebral palsy
Source: Dev Med Child Neurol. 2025 Jul 31;68(2):199–210. doi: 10.1111/dmcn.16427 (PMC12766548; doi:10.1111/dmcn.16427)
Supplement: Supplementary file 4 — Table S1: Participant characteristics by age group [file DMCN-68-199-s003.docx]

| **Supplementary Table 1: Participant characteristics by age group** | | | | | | | | |  |  |
| --- | --- | --- | --- | --- | --- | --- | --- | --- | --- | --- |
|  | **Age at assessment** | | | | | | | |  |  |
|  | **1.5-2.5 years, n=84** | | **3.0-3.5 years, n=69** | | **4.0-4.5 years, n=68** | | **5.0 years, n=100** | | **8.0-13.0 years, n=38** | |
|  | **Mean** | **SD** | **Mean** | **SD** | **Mean** | **SD** | **Mean** | **SD** | **Mean** | **SD** |
| Age (years) | 2.28 | 0.37 | 3.11 | 0.16 | 4.14 | 0.17 | 5.18 | 0.22 | 9.79 | 1.21 |
| Gestational age at birth (weeks) | 35.32 | 5.14 | 35.67 | 5.04 | 35.96 | 4.84 | 35.42 | 5.15 | 34.44 | 5.79 |
| Birth weight (grams) | 2506 | 1018 | 2638 | 1042 | 2654 | 1009 | 2516 | 1044 | 2425 | 1205 |
|  | **Frequency** | **Percent** | **Frequency** | **Percent** | **Frequency** | **Percent** | **Frequency** | **Percent** | **Frequency** | **Percent** |
| **Sex** |  |  |  |  |  |  |  |  |  |  |
| Male | 49 | 58 | 42 | 61 | 45 | 66 | 61 | 61 | 26 | 68 |
| Female | 35 | 42 | 27 | 39 | 23 | 34 | 39 | 39 | 12 | 32 |
| **GMFCS** |  |  |  |  |  |  |  |  |  |  |
| I | 37 | 44 | 35 | 52 | 29 | 43 | 40 | 40 | 19 | 50 |
| II | 10 | 12 | 3 | 4 | 12 | 18 | 21 | 21 | 9 | 24 |
| III | 17 | 20 | 11 | 16 | 10 | 15 | 15 | 15 | 5 | 13 |
| IV | 5 | 6 | 10 | 15 | 5 | 7 | 9 | 9 | 1 | 3 |
| V | 15 | 18 | 9 | 13 | 12 | 18 | 15 | 15 | 4 | 10 |
| **Motor type** |  |  |  |  |  |  |  |  |  |  |
| Unilateral spasticity | 30 | 36 | 21 | 30 | 19 | 28 | 33 | 33 | 12 | 32 |
| Bilateral spasticity | 44 | 52 | 36 | 52 | 35 | 52 | 51 | 51 | 19 | 50 |
| Other^1^ | 10 | 12 | 12 | 17 | 14 | 21 | 16 | 16 | 7 | 18 |
| **Primary feeding mode** |  |  |  |  |  |  |  |  |  |  |
| Orally fed | 75 | 89 | 61 | 88 | 57 | 84 | 87 | 87 | 32 | 86 |
| Tube fed | 9 | 11 | 8 | 12 | 11 | 16 | 13 | 13 | 5 | 14 |
| **EDACS** |  |  |  |  |  |  |  |  |  |  |
| I | 38 | 48 | 36 | 52 | 37 | 54 | 52 | 53 | 18 | 49 |
| II | 13 | 17 | 14 | 20 | 11 | 16 | 22 | 22 | 12 | 32 |
| III | 12 | 15 | 8 | 12 | 6 | 8 | 7 | 7 | 3 | 8 |
| IV | 10 | 13 | 3 | 4 | 4 | 6 | 6 | 6 | 0 | 0 |
| V | 6 | 8 | 8 | 12 | 10 | 15 | 12 | 12 | 4 | 11 |
| GMFCS: Gross Motor Function Classification System; EDACS: Eating and Drinking Classification System;^1^ Dystonic, ataxic, hypotonic or athetoid | | | | | | | | | | |
