# Supplementary material for: Brain lesion extent, growth, and body composition in children with cerebral palsy
Source: Dev Med Child Neurol. 2025 Jul 31;68(2):199–210. doi: 10.1111/dmcn.16427 (PMC12766548; doi:10.1111/dmcn.16427)
Supplement: Supplementary file 5 — Table S2: Comparison of included sample to excluded due to missing data and Australian CP register [file DMCN-68-199-s005.docx]

| **Supplementary Table 2: Comparison of included and excluded sample due to missing data at, and the Australian CP register** | | | | | | | |
| --- | --- | --- | --- | --- | --- | --- | --- |
|  | **Included current paper**  **(Growth n=124/Body comp n=115^#^)** | | **Excluded**  **(missing MRI/ growth data, n=52)^** | | **Total CP Child**  **(N=174)^** | | **Australian CP Register***  **(birth years 1993-2009)** |
|  | **Count** | **Percent** | **Count** | **Percent** | **Count** | **Percent** | **Percent** |
| **Gross Motor Function Classification System** | | | | | | | |
| GMFCS I | 50 | 40 | 22 | 44 | 72 | 41 | 35.3 |
| GMFCS II | 25 | 20 | 9 | 18 | 34 | 20 | 24.2 |
| GMFCS III | 16 | 13 | 5 | 10 | 21 | 12 | 11.8 |
| GMFCS IV | 12 | 10 | 9 | 18 | 21 | 12 | 13.3 |
| GMFCS V | 21 | 17 | 5 | 10 | 26 | 15 | 15.3 |
| **Primary motor type** | | | | | | | |
| Unilateral spasticity | 39 | 31 | 13 | 26 | 52 | 30 | 39.1 |
| Bilateral spasticity | 65 | 52 | 25 | 50 | 90 | 52 | 46.7 |
| Dystonic/ athetoid | 9 | 7 | 10 | 20 | 19 | 11 | 6.4 |
| Ataxic | 7 | 6 | 1 | 2 | 8 | 5 | 4.8 |
| Hypotonic | 4 | 3 | 1 | 2 | 5 | 3 | 3.0 |
|  | **Mean** | **SD** | **Mean** | **SD** | **Mean** | **SD** |  |
| **Height, weight, and body mass index (n=124 included n=39 excluded)** | | | | | | | |
| HZ^#^ | -0.50 | 1.37 | -0.47 | 1.12 | -0.49 | 1.37 | Not available |
| WZ^#^ | -0.45 | 1.55 | -0.15 | 1.10 | -0.38 | 1.46 |  |
| **Body composition** (**n=114 included n=30 excluded)** | | | | | | | |
| Fat free mass index^#^ | 12.05 | 1.35 | 12.20 | 1.29 | 12.08 | 1.34 | Not available |
| Fat mass index^#^ | 3.63 | 1.29 | 3.50 | 1.17 | 3.60 | 1.26 |  |
| Note: Data from last attended assessment; ^#^Children without body composition data: GMFCS I: 2, II: 1, III: 4, V: 2; Motor type: unilateral spastic: 2, bilateral spastic: 5, ataxic: 1, dystonic/athetoid: 1; ^ n=43/176 had no MRI, n=8/176 had no growth data; n=1 missing tube feeding status; GMFCS included versus excluded: Chi square statistic (df 4): 3.64, p=0.457; Motor type included versus excluded: Chi square statistic (df 5): 7.43, p=0.190; *5.8% with unknown GMFCS level and 5.0% with unknown primary motor type; ^#^ Two sample t-test with equal variances: p>0.05. | | | | | | | |
