# Supplementary material for: Brain lesion extent, growth, and body composition in children with cerebral palsy
Source: Dev Med Child Neurol. 2025 Jul 31;68(2):199–210. doi: 10.1111/dmcn.16427 (PMC12766548; doi:10.1111/dmcn.16427)
Supplement: Supplementary file 6 — Table S3: Prediction of height and weight z‐score, fat‐free mass and Fat Mass Index by brain lesion MRICS [file DMCN-68-199-s002.docx]

| **Supplementary Table 3: Mixed linear regression for height and weight Z-score, fat-free and fat mass index adjusted for MRI classification system (MRICS)** | | | | | |
| --- | --- | --- | --- | --- | --- |
|  | **Height**  **Z-score** | **Weight**  **Z-score** | **Head circumference Z-score** | **Fat free mass index** | **Fat mass**  **index** |
| Age | 0.10 | -0.02 | -0.55 | -0.80*** | 0.04 |
|  | [-0.03, 0.22] | [-0.14, 0.10] | [-1.16, 0.06] | [-1.03,-0.56] | [-0.22, 0.30] |
| Age^2^ | -0.01 | 0.00 | 0.07 | 0.05*** | 0.02** |
|  | [-0.02, 0.00] | [-0.01, 0.01] | [-0.01, 0.15] | [0.03, 0.07] | [0.00, 0.04] |
| Gestational Age | 0.86*** | 0.93*** |  |  |  |
|  | [0.24, 1.47] | [0.23, 1.64] |  |  |  |
| Gestational Age^2^ | -0.01** | -0.01** |  |  |  |
|  | [-0.02,-0.00] | [-0.02,-0.00] |  |  |  |
| Female (REF) |  |  |  |  | Ref |
| Male |  |  |  |  | -0.65*** |
|  |  |  |  |  | [-1.10,-0.19] |
| **Magnetic Resonance Imaging Classification System** | | | | | |
| Normal (REF) | Ref | Ref | Ref | Ref | Ref |
| PWMI | -0.40 | -0.63 | -0.31 | -0.31 | -0.41 |
|  | [-1.22, 0.42] | [-1.57, 0.30] | [-1.08, 0.45] | [-1.08, 0.45] | [-1.20, 0.38] |
| BM | -0.74 | -0.55 | 0.83 | -0.07 | 0.07 |
|  | [-1.98, 0.49] | [-1.96, 0.86] | [-0.89, 2.55] | [-1.20, 1.07] | [-1.10, 1.25] |
| PGMI | -0.75 | -0.67 | -0.10 | -0.10 | 0.05 |
|  | [-1.66, 0.17] | [-1.71, 0.38] | [-0.96, 0.75] | [-0.96, 0.75] | [-0.86, 0.95] |
| Miscellaneous | -0.91 | -1.05 | 0.02 | -0.68 | 0.09 |
|  | [-2.00, 0.18] | [-2.29, 0.19] | [-1.57, 1.60] | [-1.75, 0.39] | [-1.04, 1.22] |
| GA: gestational age; PWMI: predominant white matter injury; BM: brain maldevelopments; PGMI: predominant grey matter injury; M: miscellaneous; *** p<.01, ** p<.05 | | | | | |
